# The Serum IL-6 Profile and Treg/Th17 Peripheral Cell Populations in Patients with Type 1 Diabetes

**DOI:** 10.1155/2013/205284

**Published:** 2013-02-21

**Authors:** Monika Ryba-Stanisławowska, Maria Skrzypkowska, Jolanta Myśliwska, Małgorzata Myśliwiec

**Affiliations:** ^1^Department of Immunology, Medical University of Gdańsk, Dębinki 1, 80-210 Gdańsk, Poland; ^2^Clinic of Pediatrics, Department of Diabetology and Endocrinology, Medical University of Gdańsk, 80-210 Gdańsk, Poland

## Abstract

IL-6 is a pleiotropic cytokine involved in the regulation of the immune response, inflammation, and hematopoeisis. Its elevated levels are found in a range of autoimmune and chronic inflammatory diseases. IL-6 is also involved in regulation of the balance between two T cell subsets: Tregs and Th17, which have contradictory functions in the control of inflammation. The present study provides a quantitative analysis regarding the Th17/Treg cell balance in peripheral blood of children with type 1 diabetes and its association with serum IL-6 level.

## 1. Introduction

IL-6 is a pleiotropic cytokine involved in the regulation of the immune response, inflammation and hematopoeisis. Unlike many cytokines, IL-6 can be detected in the serum, although baseline levels are low in the absence of inflammation [1]. The elevated levels of IL-6 were found in autoimmune and chronic inflammatory diseases such as rheumatoid arthritis, inflammatory bowel diseases, diabetes, multiple sclerosis, and asthma [2–7]. IL-6 may also contribute to malignancies such as multiple myeloma [8] and colon cancer [9]. 

Recently it has been shown that IL-6 is involved in the regulation of a balance between two T cell subsets that play pivotal role in inflammatory and autoimmune diseases. These are IL-17-producing Th17 cells that contribute to the progression of inflammation [10, 11] and Foxp3^+^ T regulatory cells which are natural suppressors that control overactive cells [12, 13]. A balance between Th17 and Treg subsets is crucial for immune homeostasis, however it was shown to be impaired in various clinical disorders [14–20]. The balance between Tregs and Th17 is controlled by IL-6. While it favors the differentiation of Th17 cells, IL-6 inhibits the generation of Tregs [21, 22]. IL-6 exerts detrimental effects on Tregs by downregulating the expression of Foxp3 transcription factor [23, 24]. It synergizes with TGF-*β* to induce proinflammatory Th17 cells [25].

Our previous studies have shown the impaired quantitative as well as qualitative properties of Foxp3^+^ Tregs in type 1 diabetic children [26, 27]. These impairments were probably dependant on an ongoing inflammatory response in these children. In addition, we and others have shown the elevated serum IL-6 level in patients with type 1 diabetes which may play an important role in pathogenesis of diabetic microvascular complications [28–30].

Our current work shows the association between IL-6 serum level and Treg/Th17 subsets in type 1 diabetes patients. It supports the view that targeting IL-6 signaling may give benefits on the treatment of autoimmune and chronic inflammatory diseases [21].

## 2. Materials and Methods

### 2.1. Subjects

A group of 36 patients aged 14.2 (±3.6) years with long standing diabetes type 1 from the Clinic of Pediatrics, Department of Diabetology and Endocrinology, Medical University of Gdańsk was examined. The mean duration of diabetes was 7.42 (±3.94) years. Type 1 diabetes was defined according to the criteria of the American Diabetes Association [31]. Patients with microvascular complications as well as those with coexisting autoimmune, chronic and acute inflammatory diseases were excluded from the study. The control group consisted of 20 age and sex matched healthy individuals recruited during control visits in outpatient clinic. No signs of autoimmune, chronic, inflammatory, neoplastic disease at the time of sampling and no evidence of DM1 in their families was disclosed as confirmed by medical records, laboratory examination and laboratory tests. The study followed the principles of the Declaration of Helsinki and was approved by The Ethics Committee of The Medical University of Gdańsk. 

### 2.2. Sample Collection

Blood samples were immediately placed on ice, clarified by centrifugation at 3000 ×g for 5 minutes at 4°C, and kept frozen at −80°C until assayed. 

### 2.3. Blood Measurements

HbA_1_c was measured using an immunoturbidometric method using the Unimate 3 set (Hoffmann-La Roche AG, D) with a normal range of 3.0–6.0%. Fasting glucose was measured by enzymatic test (Roche Diagnostics GmbH, D). The level of C reactive protein (CRP) was measured using a high-sensitive particle-enhanced immune-turbidometric assay “Tina-quant CRP (Latex) HS” on a Roche Cobas Modular P analyser (Roche Diagnostics GmbH, D). The method was calibrated against the IFCC/CRM 470 standard. The lower detection limit was 0.03 mg/L with an assay sensitivity ranging from 0.1 to 20 mg/L. Intra-assay coefficient of variation ranged between 3.2–5.2% and interassay coefficient was 6.2–10.1%. Urinary albumin excretion was expressed as the average of three 24-hour collections obtained during 6 months prior to enrolment in the study. Micro-albuminuria was defined as albumin excretion between 30–299 mg/24 hours in at least two out of three urine samples. Urinary albumin excretion was measured by the immune-turbidometric assay using Tina-quant (Boehringer Mannheim GmbH, D).

### 2.4. Cell Isolation and Culture

Heparinised venous blood samples (4–6 mL) were collected aseptically into the tubes and used to isolate peripheral blood mononuclear cells (PBMC). 

PBMC were separated by density gradient preparation over Ficoll-Uropoline. Mononuclear cells at the interface were carefully transferred into a Pasteur Pipette, then treated with RBC Lysis Buffer (BioLegend, USA) and washed twice in PBS. 

For Th17 analysis cells were suspended at a density of 2 × 10^6^ cells/mL and cultured in RPMI 1640 supplemented with 5% heat-inactivated fetal calf serum (FCS). Cultures were stimulated with 50 ng/mL of phorbol myristate acetate—PMA (Sigma, USA) plus 1 *μ*L/mL of ionomycin (Sigma, USA) for 4 h in the presence of 1 *μ*L/mL of monensin (BioLegend, USA). After 4 hours of culture in 37°C with 5% CO_2_ the contents of the wells were transferred to 5 mL polystyrene round bottom test tubes (BD Bioscience, USA) and centrifuged at 200 ×g for 5 minutes. 

For Treg analysis, fresh, resting PBMCs were suspended in 5 mL polystyrene round bottom test tubes (BD Bioscience, USA) at a density of 1 × 10^6^ cells per 1 mL of RPMI 1640 and centrifuged at 200 ×g for 5 minutes. 

Cell pellets were then destined for flow cytometric staining.

### 2.5. Flow Cytometric Staining and Analysis

Before staining cells were washed with Cell Staining Buffer (BioLegend, USA). Cells were stained with anti-CD4 antibody (IgG1, *κ* mouse Pe/Cy5, Clone RPA-T4, BioLegend, USA). After 20 minutes incubation at room temperature, cells were washed and stained for intracellular expression of Foxp3 in case of Treg and IL-17A in case of Th17 cells. The following monoclonal antibodies were used for Treg and Th17 intracellular staining, respectively: anti-Foxp3 (IgG1, *κ* mouse Alexa-Fluor 488, Clone 206D, BioLegend, USA) and anti-IL17A (IgG1, *κ* mouse FITC, Clone BL168, BioLegend, USA). Intracellular staining for Foxp3 and IL-17A was performed with ready-to-use kits according to the manufacturers suggestions (BioLegend, USA). Expression of cell surface and intracellular markers was assessed using flow cytometry (LSRII, Becton Dickinson, USA) after gating on live lymphocytes according to forward and side scatter. Positive signal for each staining was established using appropriate isotype controls. The expression of Foxp3 in the CD4^+^Foxp3^+^ as well as expression of IL17-A in the CD4^+^IL-17A^+^ gates were quantified by determining mean fluorescence intensity (MFI). It was quantified as a ratio of mean fluorescence intensity for Foxp3 or IL-17A to MFI for appropriate isotype control. Data were analyzed by FACSDiva 6.0 Software (Becton Dickinson, USA). 

### 2.6. Determination of IL-6 Level

Serum level of IL-6 was measured by the ultrasensitive immunoenzymatic ELISA method (Quantikine High Sensitivity Human IL-6 kit, R&D Systems Inc, USA) according to the manufacturer protocol. Minimum detectable concentrations were determined by the manufacturer as 0.03 pg/mL.

### 2.7. Statistical Analysis

All statistical analyses were performed using Statistica 8.0 (StatSoft, Inc USA). 

The differences between the groups were calculated with the nonparametric *U* Mann Whitney tests. Spearman's correlations were used to compare cell frequencies with analyzed parameters. *P* values ≤0.05 were considered statistically significant.

## 3. Results

### 3.1. General Characteristics of Each Analyzed Group

DM1 patients had higher values of HbA_1_c, CRP as well as serum level of IL-6 in comparison to the age and sex-matched healthy young individuals from the control group (Table 1).

### 3.2. CD4^+^Foxp3^+^ Regulatory T Cells in Peripheral Blood of DM1 Patients

**Figure 1:**
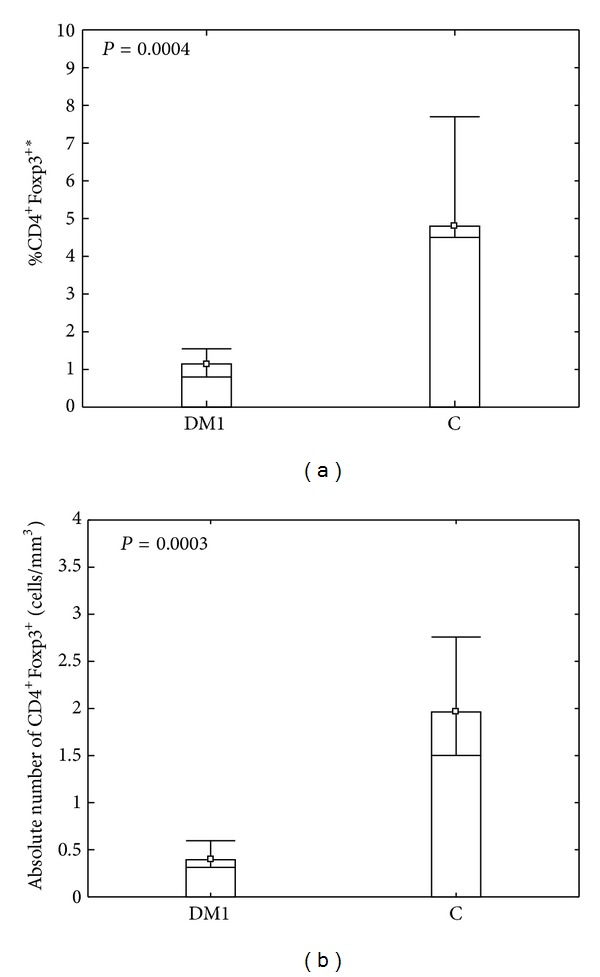
Comparison between percentage (a) and absolute number (b) of CD4^+^Foxp3^+^ T cells in peripheral blood of diabetic type 1 patients and control group. Fresh, resting PBMCs from diabetic type 1 patients (DM1) and healthy individuals (C) were stained with antibodies against CD4 and Foxp3 molecules and analyzed using flow cytometry. The gate was set on CD4^+^ lymphocytes according to forward scatter and staining with CD4-Pe/Cy5 and then the percentage of CD4^+^Foxp3^+^ T cells in CD4^+^ gate was determined. Data were calculated with *U* Mann Whitney test and presented as medians and minimum/maximum. The median percentage of CD4^+^Foxp3^+^ T regulatory cells in DM1 and control group was 1.15 (0.6/3.0) and 4.8 (3.8/8.8), respectively. The median absolute number of CD4^+^Foxp3^+^ T regulatory cells in DM1 and control group was 0.39 (0.21/1.2) and 1.96 (1.3/3.43), respectively. *The percentage of cells among peripheral blood lymphocytes.

The analysis of Tregs in peripheral blood of DM1 patients and healthy individuals revealed lower percentage and the absolute number of CD4^+^Foxp3^+^ regulatory T cells in diabetic type 1 patients in comparison to healthy individuals from the control group (Figure 1; *P* = 0.0004 and *P* = 0.0003, resp.). Similar results were found when analyzing the expression of Foxp3 in CD4^+^Foxp3^+^ gate, however this was not statistically significant (*P* = 0.08).

### 3.3. CD4^+^IL17A^+^ Th17 Cells in Peripheral Blood of DM1 Patients

**Figure 2:**
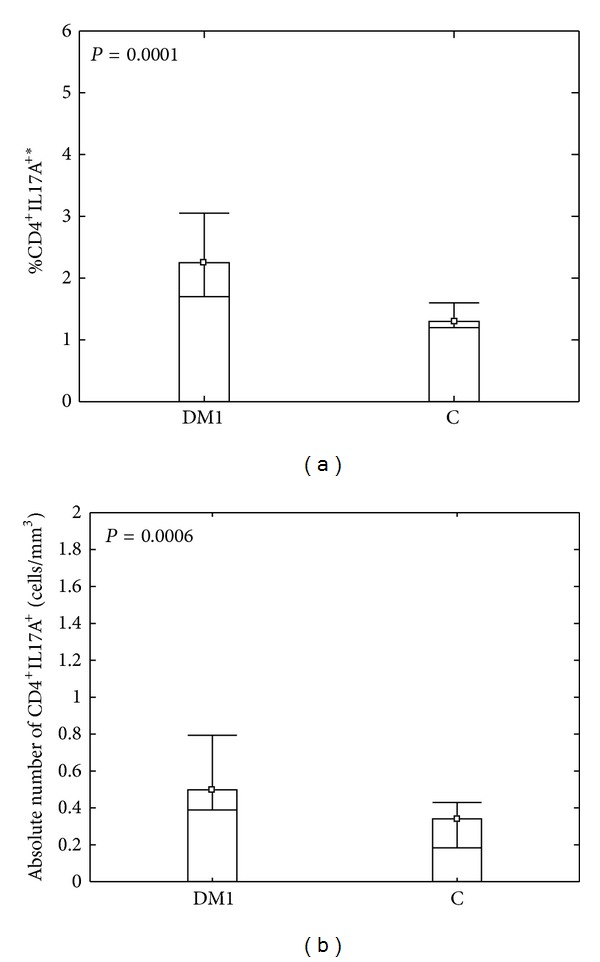
Comparison between percentage (a) and absolute number (b) of CD4^+^IL17A^+^ T cells in peripheral blood of diabetic type 1 patients and control group. PBMCs from diabetic type 1 patients (DM1) and healthy individuals (C) were cultured and stimulated as described in Section 2 and then stained with antibodies against CD4 and IL17A. The percentage of CD4^+^IL17A^+^ T cells was determined by flow cytometry. Analyzing CD4^+^IL17A^+^ cells, dot plots representing anti-CD4 versus SS were carried out to establish CD4^+^ and CD4^−^  lymphocyte gates. Then, the anti-CD4 versus IL17A from CD4^+^ gate dot plot was generated and the frequency of Th17 cells was determined. Data were calculated with *U* Mann Whitney test and presented as medians and minimum/maximum. The median percentage of CD4^+^IL17A^+^ T cells in DM1 and control group was 2.25 (0.8/5.4) and 1.3 (0.3/2.9), respectively. The median absolute number of CD4^+^IL17A^+^ T cells in DM1 and control group were 0.49 (0.9/1.8) and 0.34 (0.06/0.59), respectively. *The percentage of cells among peripheral blood lymphocytes.

Th17 immunity is associated with inflammatory and autoimmune diseases. We, therefore, analyzed this cell subset in peripheral blood of type 1 diabetic patients and healthy individuals. When comparing CD4^+^IL17A^+^ cell numbers as well as the expression of IL17A among CD4^+^IL17A^+^ cells between DM1 and healthy group, we found that diabetic type 1 patients had higher frequency as well as the absolute number of CD4^+^IL17A^+^ Th17 cells than their healthy counterparts (Figure 2; *P* = 0.0001 and *P* = 0.0006, resp.). As to the expression of IL17A defined as mean fluorescence intensity we found statistically significant difference between analyzed groups. CD4^+^IL17A^+^ cells from DM1 patients showed higher expression of IL17A than Th17 cells from the control group (*P* = 0.03).

### 3.4. The Association of IL-6 Serum Level with Treg/Th17 Peripheral Counts

As it was mentioned, IL-6 is the cytokine that has impact on Tregs as well as Th17 cells. In addition, the elevated level of this cytokine was observed by other authors in type 1 diabetic individuals [32, 33].

**Figure 3:**
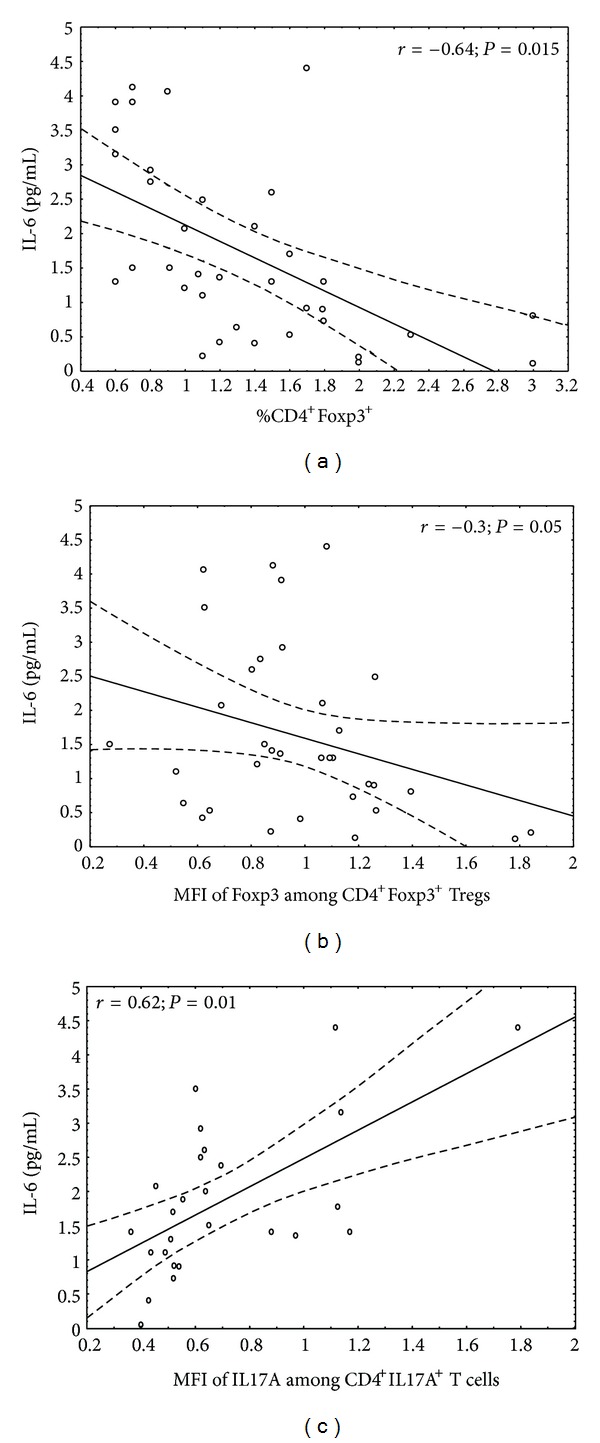
Association of IL-6 serum level with the status of Tregs/Th17 in analyzed DM1 subjects. The level of IL-6 was measured in the blood of DM1 children and correlated with the peripheral status of Tregs and Th17 cells. The Spearman test was used to calculate the strength of correlation. (a) The correlation between CD4^+^Foxp3^+^ frequency and IL-6 serum level in DM1 subjects (*n* = 34; *r* = [−0.64]; *P* = 0.015); (b) the correlation between mean fluorescence intensity (MFI) of Foxp3 among CD4^+^Foxp3^+^ Tregs and IL-6 serum level in DM1 subjects (*n* = 34; *r* = [−0.3]; *P* = 0.05); (c) the correlation between mean fluorescence intensity (MFI) of IL17A among CD4^+^IL17A^+^ T cells and IL-6 serum level in DM1 subjects (*n* = 26; *r* = 0.62; *P* = 0.01).

When we analyzed our groups we found that the serum IL-6 concentrations were about five times higher in the patients than in the healthy controls. Interestingly, we found negative, statistically significant correlation between serum IL-6 level and frequency of CD4^+^Foxp3^+^ T cells (Figure 3(a), *r* = [−0.64]; *P* = 0.015). The expression of Foxp3 among CD4^+^Foxp3^+^ Treg cells in DM1 patients also significantly correlated with IL-6 serum level (Figure 3(b), *r* = [−0.3]; *P* = 0.05). As to Th17 cells in diabetic group, we found positive correlation between this cell subset and serum IL-6 level, however this was statistically significant only when the MFI of IL17A among CD4^+^IL17A^+^ T cells was taken into account (Figure 3(c), *r* = 0.62; *P* = 0.01).

## 4. Discussion

Our work shows the dysregulated balance of Th17 and Tregs in patients with type 1 diabetes, which may partly depend on impaired IL-6 signalization. The previously published data on serum IL-6 level in type 1 diabetes are contradictory. Some authors have reported lower or normal levels of this cytokine [34, 35], but the majority of papers link type 1 diabetes with higher IL-6, which is thought to be associated with prior hyperglycemia and/or progression of microvascular diabetic complications [5, 28–30, 32, 33]. The latter are supported by our results, as we found the higher level of this cytokine in serum of diabetic patients in comparison to healthy controls. IL-6 was found to be associated with lower frequency of CD4^+^Foxp3^+^ Tregs as well as lower intensity of Foxp3 expression in these cells. On the other hand, the level of IL-6 was in positive relation with IL17A produced by Th17 cells. Interleukin 6 acts via membrane-bound and soluble receptors. The classical IL-6 signaling involves binding to IL-6R receptor on different cell types. Moreover, cells that do not express IL-6R may be also activated by IL-6 when it binds to soluble form of the receptor (sIL-6R) in a mechanism known as trans-signalization [36]. IL-6 trans-signaling via soluble IL-6 receptor blocks the expression of Foxp3 which correlates with loss of Tregs suppressive function [37]. One group has shown significantly higher level of sIL-6R in the serum as well as vitreous fluid of patients with proliferative diabetic retinopathy in comparison to non-diabetic group [38], but it would be reasonable to investigate the level of this protein in the context of Treg/Th17 cells. Recently, the studies on mice as well as on patients with rheumatoid arthritis showed that blocking IL-6 receptor with monoclonal antibody resulted in decrease in the percentage of Th17 cells and an increase in the percentage of Treg cells [39, 40].

In conclusion, one may suspect that the higher level of IL-6 seen in type 1 diabetic patients affects the quantitative and qualitative features of Treg/Th17 shifting the balance towards inflammatory Th17 cells. Interestingly, the positive correlation between IL-6 and TNF-*α* level in DM1 patients was found [39], and TNF-*α* may impair the Treg subset which was previously shown by us [26]. Taken together the above results, we suggest an important regulatory role of IL-6 in the progression of diabetes and its complications. Future studies are needed to show if blockade of IL-6 signaling has beneficial effect on Treg subsets in type 1 diabetic patients. 

## Figures and Tables

**Table 1:** Basic characteristic of each analyzed group.

Group	Age, mean (SD) years	Disease duration, mean (SD) years	HbA_1_c, mean (SD) %	Albumin excretion rate, mean (SD) mg/24 h	CRP, median (IQR)** mg/L	Serum IL-6, median (IQR)** pg/mL
DM1 (*n* = 36)	14.2 (3.6)	7.42 (3.94)	8.8 (1.96)	17.7 (5.03)	1.94 (1.08/3.07)	1.6 (1.3/2.6)
Healthy (*n* = 20)	17.6 (1.3)	—	4.01 (0.13)	—	0.62 (0.37/1.04)	0.29 (0.23/0.47)
P*	0.31		0.003		0.02	0.001

*The significance between DM1 patients and healthy individuals, SD: Standard Deviation, IQR: Interquartile Range, **Interquartile range represents the 25th and 75th percentiles.

## Abbreviations

IL: Interleukin
TGF: Transforming growth factor
DM1: Diabetes mellitus 1
Treg: Regulatory T cells
MFI: Mean fluorescent intensity
TNF: Tumor necrosis factor.
